# Late Medicaid expansion was associated with higher Medicaid coverage: source-specific evidence from Oklahoma, Missouri, and North Carolina

**DOI:** 10.3389/fpubh.2026.1942538

**Published:** 2026-10-08

**Authors:** Xinying Lu

**Affiliations:** Department of Public Policy, UCLA Luskin School of Public Affairs, University of California, Los Angeles, Los Angeles, CA, United States

**Keywords:** American Community Survey, crowd-out, difference-in-differences, direct-purchase insurance, health insurance coverage, Medicaid, Medicaid expansion, randomization inference

## Abstract

**Background:**

Evidence remains limited on Medicaid expansions implemented after 2020 and on whether increases in Medicaid coverage were accompanied by changes in specific insurance sources.

**Methods:**

This repeated cross-sectional study used 2015–2024 American Community Survey's 1-year public use microdata sample data for adults ages 18–64. Weighted state-year difference-in-differences models compared the 2021 expansions in Oklahoma and Missouri and the 2023 expansion in North Carolina with ten states that had not implemented full ACA Medicaid expansion through 2024. Models included state and year fixed effects. Conventional state-clustered 95% confidence intervals were supplemented with exact state-level randomization inference. Medicaid, uninsurance, employer-sponsored insurance, direct-purchase coverage, Medicare, other public coverage, and multiple coverage were estimated separately.

**Results:**

Medicaid coverage increased by 3.389 percentage points in Oklahoma–Missouri (95% CI, 1.024 to 5.754; exact randomization-inference *P* = 0.030) and by 3.332 points in North Carolina (95% CI, 2.823 to 3.840; *P* = 0.091). The largest estimates occurred among adults below 138% of the federal poverty level. In Oklahoma–Missouri, direct-purchase coverage declined by 1.968 points (95% CI, −2.693 to −1.244; *P* = 0.015), while employer-sponsored insurance and multiple coverage showed no corresponding assignment-based evidence of change. The North Carolina direct-purchase estimate was not supported by full-period exact randomization inference (*P* = 0.182). The Oklahoma–Missouri Medicaid estimate increased from 1.880 points in 2021–2022 to 4.887 points in 2023–2024 and changed little after adjustment for measured Medicaid-unwinding indicators. Synthetic estimates were similar to the conventional DID estimates, although pre-expansion diagnostics and treatment-specific-trend models indicated sensitivity to the assumed counterfactual trend.

**Conclusions:**

Late Medicaid expansion was associated with increased Medicaid coverage, particularly among adults below 138% of the federal poverty level. The Oklahoma and Missouri findings were consistent with partial substitution away from direct purchase coverage and a larger Medicaid coverage association in later years. The limited number of treated states and concerns about trends before expansion warrant cautious interpretation.

## 1 Introduction

Medicaid is a central component of the United States health insurance system and an important source of coverage for adults with low incomes. In 2024, Medicaid spending reached $931.7 billion, accounting for 18% of national health expenditures (1). In January 2026, approximately 75.3 million people were enrolled in Medicaid or the Children's Health Insurance Program, including about 68.0 million enrolled in Medicaid (2). As Medicaid covers a large population and represents a substantial share of health care spending, changes in eligibility have implications for insurance access, public finance, and the distribution of coverage across public and private sources.

The Affordable Care Act (ACA) expanded Medicaid eligibility to adults with family incomes up to approximately 138% of the federal poverty level (3). Although the expansion began in 2014, states did not adopt the policy simultaneously. The Supreme Court's 2012 decision in *National Federation of Independent Business v Sebelius* made expansion optional for states. Consequently, some states implemented expansion during the initial ACA period, others adopted it several years later, and some had not implemented full ACA Medicaid expansion by 2024 (4, 5). This staggered adoption created policy variation that can be examined using quasi-experimental comparisons of within-state coverage changes following expansion with contemporaneous changes in states that had not implemented full expansion.

Most research on Medicaid expansion has focused on states that implemented the policy during the initial ACA period. Studies of these early expansions generally found increases in Medicaid enrollment and overall insurance coverage, reductions in uninsurance, and relatively limited effects on labor supply (6–9). Evidence on later-adopting states remains highly limited. Evaluations of Virginia's 2019 expansion and parental expansions implemented after 2015 found increases in public coverage and improvements in aspects of family financial wellbeing (10, 11). However, population-level evidence on expansions implemented after 2020, such as how coverage changed across specific insurance sources, remains comparatively limited.

Late-expansion states entered expansion under insurance-market, Medicaid-enrollment, and administrative conditions that differed from those facing states that expanded around 2014. Their implementation periods overlapped with the pandemic-era Medicaid continuous-enrollment policy and the subsequent resumption of eligibility redeterminations, commonly referred to as Medicaid unwinding. These developments produced substantial cross-state variation in Medicaid enrollment, disenrollment, and administrative implementation (12–14). The experiences of the initial expansion states therefore may not fully represent coverage changes associated with expansion in this more recent policy environment.

The policy implications of increased Medicaid coverage depend partly on the accompanying changes in other coverage categories. At the population level, an increase in Medicaid coverage accompanied by lower uninsurance is consistent with a net expansion of insurance coverage. An increase in Medicaid coverage accompanied by a reduction in private coverage is instead consistent with substitution between public and private insurance. When expanded public eligibility causes private insurance to be replaced by Medicaid, this substitution is commonly described as crowd-out. Employer-sponsored insurance and direct-purchase coverage are examined here as separate forms of private coverage, whereas Medicare and other public insurance are not treated as private-insurance crowd-out. Prior studies have reported mixed evidence regarding reductions in private coverage following Medicaid expansion (15, 16). Direct-purchase coverage may include both marketplace and non-marketplace plans, and respondents may report more than one insurance source. Separating these outcomes is therefore necessary to identify which components of coverage changed, although population-level changes cannot establish that the same individuals moved from one insurance source to another.

Coverage changes may also differ across population groups. Adults with family incomes below 138% of the federal poverty level are most directly targeted by ACA Medicaid expansion and may therefore experience the largest coverage changes. Business owners and self-employed workers may have less stable access to employer-sponsored insurance and may be particularly sensitive to changes in public insurance eligibility. Young adults likewise tend to experience less stable insurance coverage. Existing research indicates that Medicaid expansion improved insurance coverage and access among adults with low incomes, self-employed workers, and young adults, particularly those who were socioeconomically disadvantaged (17–19). Examining these populations separately helps determine whether average coverage changes conceal important differences across groups.

This study used American Community Survey 1-year Public Use Microdata Sample data from 2015 to 2024 and had three objectives: (1) to estimate changes in Medicaid coverage following late expansion relative to contemporaneous changes in comparison states; (2) to examine accompanying changes in uninsurance and specific insurance sources, such as employer sponsored insurance, direct purchase coverage, Medicare, other public coverage, and multiple coverage; and (3) to estimate Medicaid coverage changes among adults below 138% of the federal poverty level, business owners, and adults ages 18 to 35. Additional analyses examined changes across periods after expansion and sensitivity to Medicaid unwinding and alternative counterfactual trends.

## 2 Materials and methods

### 2.1 Data sources and study sample

This repeated cross-sectional, quasi-experimental study pooled the 2015–2024 American Community Survey (ACS) 1-year Public Use Microdata Sample (PUMS) person files (20). The ACS is an annual survey conducted by the U. S. Census Bureau and provides information on state of residence, demographic and socioeconomic characteristics, and current health insurance coverage by source. The study period began in 2015 to provide multiple pre-expansion years for assessing baseline coverage trends. It ended in 2024 because it was the most recent ACS 1-year PUMS available when the analysis was conducted and the first full post-expansion year for the latest expansion examined.

Pandemic-related data-collection disruptions prevented the release of standard 2020 ACS 1-year estimates. The Census Bureau instead released an experimental 2020 ACS 1-year PUMS with specially constructed person weights (21). The primary specification retained 2020 using the supplied experimental person weight. All principal analyses were repeated after excluding 2020 as a sensitivity analysis.

The ACS data were supplemented with the Centers for Medicare & Medicaid Services (CMS) State Medicaid and Children's Health Insurance Program (CHIP) Eligibility Processing Data (22). This monthly, state-reported administrative dataset provides information on eligibility renewals and disenrollments during the resumption of Medicaid and CHIP eligibility redeterminations in 2023 and 2024. The construction and use of the state-year unwinding measures are described in Section 2.5.

The primary study population included adults aged 18–64 residing in the treatment and comparison states defined in Section 2.3. The age restriction focused the analysis on the nonelderly adult population principally affected by the ACA Medicaid expansion, whereas children and adults aged 65 or older are subject to different public insurance eligibility structures. Records were retained when age, state, survey year, and the ACS person weight were valid, and the person weight was positive. After these restrictions, the primary 13-state sample contained 6,190,223 unweighted person-level observations. Treatment-specific and subgroup sample sizes are reported in the corresponding tables.

### 2.2 Measures and analytic groups

Insurance outcomes were constructed directly from the ACS coverage-source indicators. Employer- or union-sponsored insurance was identified using HINS1, direct-purchase coverage using HINS2, Medicare using HINS3, Medicaid using HINS4, and other public coverage using HINS5 or HINS6. Other public coverage therefore included TRICARE or other military coverage and Department of Veterans Affairs coverage. Indian Health Service coverage, reported through HINS7, was not treated as comprehensive health insurance. Uninsurance was defined as HICOV = 2, and any health insurance indicated at least one comprehensive coverage source.

Each insurance source was coded as a separate binary outcome. The source-specific indicators were permitted to overlap; for example, a respondent reporting both Medicaid and employer-sponsored insurance contributed to both corresponding outcomes. Multiple coverage equaled 1 when a respondent reported at least two of the six comprehensive insurance sources represented by HINS1–HINS6.

The composition of direct-purchase coverage was examined further using HIMRKS, the ACS Marketplace premium subsidy indicator available beginning in 2019. Among respondents reporting direct-purchase coverage, subsidized marketplace coverage was defined as HINS2 = 1 and HIMRKS = 1. Direct-purchase coverage without a premium subsidy was defined as HINS 2= 1 and HIMRKS = 2. This category may include both unsubsidized Marketplace plans and plans purchased outside the marketplace because the ACS does not distinguish between them. Residual direct-purchase coverage was defined as HINS2 = 1 and HIMRKS = 0.

Medicaid coverage was also examined in three policy-relevant groups. Business owners were identified from the ACS class-of-worker coding for respondents who were self-employed in their own incorporated or unincorporated business, professional practice, or farm. Adults below 138% of the federal poverty level were identified using POVPIP < 138, which measures family income relative to the applicable poverty threshold. The young-adult analysis was restricted by age to respondents aged 18–35.

### 2.3 Treatment and comparison states

Two treatment designs were estimated separately. Oklahoma and Missouri were pooled as the 2021 expansion cohort. The years 2015–2020 were defined as the pre-expansion period, and 2021–2024 were defined as the post-expansion period. As both expansions began during 2021, the 2021 annual observation represents partial-calendar-year exposure. North Carolina implemented Medicaid expansion on 1 December 2023. Accordingly, 2024 was defined as its first full post-expansion year. The predominantly pre-expansion 2023 survey year was retained as a transition year in the primary North Carolina specification and was excluded entirely in a transition-year sensitivity analysis.

South Dakota also implemented Medicaid expansion during the study period but was not analyzed as a separate treatment case because its ACS sample was substantially smaller than those of Oklahoma, Missouri, and North Carolina. The findings therefore concern the treatment cases examined and should not be interpreted as estimates for every late-expansion state.

The comparison group consisted of the ten states that had not implemented full ACA Medicaid expansion through 2024: Alabama, Florida, Georgia, Kansas, Mississippi, South Carolina, Tennessee, Texas, Wisconsin, and Wyoming (5). These states were used to estimate contemporaneous coverage changes in the absence of the late expansions examined here. Wisconsin differed from the other comparison states because it covered adults with incomes up to 100% of the federal poverty level through a waiver. Wisconsin was retained in the primary comparison group under the predefined full-expansion classification, and both treatment designs were re-estimated after removing Wisconsin as a sensitivity analysis.

### 2.4 Primary statistical analyses

Individual ACS records were used to calculate PWGTP-weighted coverage rates for each state and survey year. Each state-year observation was then weighted in the regression by the sum of PWGTP in that cell. The first use of PWGTP made each state-year coverage rate representative of the corresponding population, while the second allowed state-year cells representing larger populations to receive proportionately greater weight in the regression.

For insurance outcome *k*, state *s*, and year *t*, the primary weighted state-year difference-in-differences (DID) model was:


$$\begin{array}{l} {Ȳ}_{st}^{k}=\alpha +\beta_{k}\left(Treat_{s}\times post_{t}\right)+\mu_{s}+\tau_{t}+\epsilon_{st}^{k} \end{array}$$ (1)

In Equation 1, the outcome is the PWGTP-weighted state-year rate for insurance outcome *k*. The treatment indicator identifies the treatment state or states in the corresponding design, and the post-expansion indicator identifies the applicable post-expansion period. The coefficient on their interaction is the DID estimate: the post-expansion change in the treatment states relative to the contemporaneous change in the comparison states. State fixed effects account for time-invariant differences across states, and year fixed effects account for changes common to all states. Coefficients were multiplied by 100 and reported as percentage-point changes.

The Oklahoma–Missouri and North Carolina designs were estimated separately because they had different expansion dates and different amounts of post-expansion follow-up. The primary specification included state and year fixed effects without individual-level covariates. This specification targeted changes in population-level coverage while avoiding conditioning the principal estimate on characteristics that might themselves respond to Medicaid expansion.

Equation 1 was estimated separately for each insurance outcome defined in Section 2.2. Medicaid coverage was estimated in the full sample and in the three policy-relevant groups. The PWGTP-weighted prevalence of multiple coverage was also summarized before and after expansion in the treatment and comparison groups.

The HIMRKS decomposition applied Equation 1 separately to subsidized Marketplace coverage, direct-purchase coverage without a premium subsidy, and residual direct-purchase coverage. As HIMRKS was first available in 2019, this supplementary analysis used the 2019–2024 period. Its primary specification retained 2020 to remain consistent with the primary study design, and a sensitivity specification excluded 2020. The shorter window provided only two Oklahoma–Missouri pre-expansion years when 2020 was retained and one pre-expansion year when 2020 was excluded; the HIMRKS estimates were therefore treated as supplementary evidence about the composition of direct-purchase coverage.

Conventional 95% confidence intervals for the DID estimates were calculated from standard errors clustered by state, using a *t* distribution with *G-1* degrees of freedom, where *G* is the number of states in the corresponding design. As each design contained only one or two treated states, conventional cluster-robust hypothesis tests could provide unreliable inference (23, 24). Exact state-level randomization inference was therefore used for the primary DID analyses.

The number of treated states and the policy timing were held fixed, treatment status was reassigned among the analytic states, and the complete corresponding model was re-estimated for every possible assignment. The two-sided exact randomization-inference p-value was calculated as shown in Equation 2:


$$\begin{array}{l} P_{RI}=\frac{1}{\left|A\right|}\Sigma_{a\epsilon A}I\left(\left|{\hat{\beta }}_{a}|\geq |{\hat{\beta }}_{obs}\right|\right) \end{array}$$ (2)

For the Oklahoma–Missouri design, the reassignment set contained all 66 possible ways of selecting two treated states from the 12 analytic states. The minimum attainable exact *P* value was therefore 1/66 = 0.015. For the North Carolina design, the reassignment set contained all 11 possible ways of selecting one treated state from the 11 analytic states. The minimum attainable exact *P* value was therefore 1/11 = 0.091. Consequently, the North Carolina design could not produce an exact *P* value below 0.05, even if the observed estimate was the most extreme estimate in the reassignment distribution. The term “exact” refers to complete enumeration under this procedure; it does not imply that states adopted Medicaid expansion at random.

Unless otherwise specified, the strength of evidence for the primary DID results was assessed using exact randomization-inference *P* values. Conventional state-clustered 95% confidence intervals were retained to describe the magnitude and conventional uncertainty of the estimates. The tables identify the source of inference used for each analysis. All reported tests were two-sided.

### 2.5 Pre-expansion trends and robustness analyses

The diagnostic and sensitivity analyses followed four stages. First, event-study models and joint tests assessed pre-expansion coverage patterns. Second, treatment-specific trends and synthetic methods examined alternative constructions of the counterfactual coverage path. Third, policy timing and Medicaid unwinding analyses evaluated changes across post-expansion periods and differences in eligibility-redetermination activity. Finally, the sensitivity analyses varied the included ACS years, comparison-state composition, transition-year treatment, and demographic adjustment.

Pre-expansion Medicaid coverage patterns were assessed using the event-study model specified in Equation 3, which replaced the single post-expansion interaction in Equation 1 with treatment-by-year indicators:


$$\begin{array}{l} {Ȳ}_{st}=\alpha +\Sigma_{y\neq y0}\delta_{y}\left[Treat_{s}\times I\left(t=y\right)\right]+\mu_{s}+\tau_{t}+\;\epsilon_{st} \end{array}$$ (3)

The omitted reference year was 2019 for Oklahoma–Missouri, the final standard ACS year before the experimental 2020 file and the 2021 expansions. North Carolina used 2022, its final complete pre-expansion year before the 2023 transition period. For Oklahoma–Missouri, the joint pre-expansion test included all years before 2021 except the 2019 reference year. For North Carolina, the joint test included 2015–2021. The 2023 observation was retained in the North Carolina event-study model as a transition year but was not included among the pre-expansion coefficients tested jointly. Conventional state-clustered *F* tests evaluated whether the relevant pre-expansion interaction coefficients were jointly equal to zero (25). These tests were interpreted as diagnostics of the parallel-trends assumption rather than proof that the assumption held.

As the event-study diagnostics indicated potential differences in pre-expansion trends, Medicaid coverage was re-estimated using two alternative approaches to the counterfactual trend. First, a treatment-specific linear time trend was added to Equation 1. The trend was specified as the treatment indicator interacted with survey year minus 2015; subtracting 2015 centered the time variable without changing the estimated slope. As a treatment-specific trend can also absorb a genuine policy effect that develops gradually, this specification was treated as a sensitivity analysis rather than the preferred estimator. Its confidence intervals and *P*-values were based on conventional state-clustered inference; exact randomization inference was not calculated for this specification.

Second, traditional synthetic control and synthetic difference-in-differences were used to construct weighted comparison series that more closely reproduced the pre-expansion Medicaid coverage paths of the treatment cases (26, 27). For Oklahoma–Missouri, the treated series was the equal-weighted mean of the two states' annual Medicaid coverage rates. This differs from the population-weighted state-year structure of the primary DID and was therefore treated as an alternative estimand. For North Carolina, the treated series was the state's annual Medicaid coverage rate. The ten comparison states formed the donor pool.

Traditional synthetic control selected donor-state weights to reproduce the pre-expansion treated trajectory. Synthetic difference-in-differences estimated both donor-state weights and pre-expansion time weights. Both approaches were estimated with and without the experimental 2020 ACS. Assignment-based exact *P* values were calculated by applying the corresponding synthetic procedure to all 66 possible two-state assignments for Oklahoma–Missouri and all 11 possible single-state assignments for North Carolina. Conventional state-clustered confidence intervals were not applied to the synthetic estimators. The North Carolina synthetic estimates were treated as supplementary because only one complete post-expansion year was available.

Post-expansion timing was examined by replacing the Oklahoma–Missouri post-expansion interaction with separate interactions for 2021–2022 and 2023–2024. Each coefficient compared the specified post-expansion period with the common 2015–2020 pre-expansion period. Exact randomization-inference *p*-values were calculated separately for the two period-specific coefficients. Their difference was tested directly using conventional state-clustered inference because exact randomization inference was not calculated for the late-minus-early contrast. For North Carolina, the primary specification retaining 2023 as a transition year was compared with a specification that removed 2023 entirely.

The Medicaid-unwinding analysis had three components. First, state-level unwinding measures were compared descriptively across the treatment and comparison states. Second, the Oklahoma–Missouri analysis distinguished 2021–2022, before the resumption of eligibility redeterminations, from 2023–2024. Third, each unwinding measure was added separately to the weighted state-year DID model.

Three state-year unwinding measures were constructed from the CMS monthly counts. Total disenrollment among completed renewal outcomes was calculated as the number disenrolled divided by the sum of renewals and disenrollments. Procedural disenrollment among completed renewal outcomes used the same denominator but counted terminations occurring because the state lacked sufficient information to complete the eligibility determination. Ex parte renewal among renewed beneficiaries was calculated as renewals completed using available administrative information, without requiring a renewal form, divided by all renewals.

These definitions followed the CMS Medicaid and CHIP Eligibility Processing Data reporting specifications (28).

Monthly numerators and denominators were summed within each state-year before the annual rates were calculated; the annual measures were not averages of monthly percentages. The analytic extract covered April–December 2023 and January–August 2024, corresponding to the complete reporting window available in the archived data extract used for this analysis. For descriptive comparisons, group summaries were calculated as unweighted means of the corresponding state-level rates, and individual state values were also reported.

Each unwinding measure was set to zero before 2023 and entered into a separate DID model. The measures were not included simultaneously because total and procedural disenrollment are mechanically related, all three measures characterize different parts of the same renewal process, and the number of analytic states was limited. Exact state-level randomization inference was recalculated for each unwinding-adjusted model.

CMS notes that North Carolina's reported renewal outcomes cover initiated renewals scheduled for completion and do not include renewals that the state was unable to initiate by the end of the reporting month. The North Carolina unwinding measures were therefore interpreted with additional caution.

Additional sensitivity analyses excluded the experimental 2020 ACS and removed Wisconsin from the comparison group. When Wisconsin was removed, the treatment-assignment distributions were recalculated using the reduced sets of states. The Oklahoma–Missouri design then contained 55 possible two-state assignments, and the North Carolina design contained 10 possible single-state assignments.

Finally, a separate individual-level Medicaid model evaluated sensitivity to demographic composition. This model used PWGTP weights, state and year fixed effects, and the treatment-by-post-expansion interaction, while including only age, age squared, female sex, Black race, Hispanic or Latino ethnicity, Native American race, Asian race, and immigrant status. The complete covariate-adjusted model was re-estimated for all 66 Oklahoma–Missouri assignments and all 11 North Carolina assignments to obtain exact two-sided randomization-inference *P*-values.

## 3 Results

### 3.1 Study population and descriptive coverage patterns

Table 1 summarizes the study population in the 13 analytic states during the period from 2015 to 2024. Any-insurance coverage was 84.276% in the pooled Oklahoma–Missouri sample and 85.091% in North Carolina, compared with 81.356% in the comparison states. The samples also differed in insurance sources and several demographic and socioeconomic characteristics, which are reported in Table 1.

**Table 1:** Weighted descriptive characteristics of adults aged 18–64 in analytic states, 2015–2024.

Variable	Combined analytic states	Oklahoma and Missouri	North Carolina	Comparison states
Panel A. Coverage outcomes				
Any health insurance, %	81.974	84.276	85.091	81.356
Medicaid, %	10.653	10.874	11.890	10.484
No insurance, %	18.026	15.724	14.909	18.644
Employer-sponsored insurance, %	58.614	61.696	59.125	58.214
Direct-purchase coverage, %	12.822	11.184	13.289	12.948
Medicare, %	3.855	4.787	4.240	3.707
Other public coverage, %	4.740	4.555	6.343	4.573
Multiple coverage, %	8.083	8.193	9.078	7.955
Panel B. Subgroups				
Business owner, %	9.419	8.592	8.829	9.579
Below 138% FPL, %	18.790	19.341	18.874	18.719
Ages 18–35, %	39.730	40.168	39.299	39.731
Panel C. Socioeconomic characteristics				
Age, mean	40.584	40.574	40.712	40.570
Years of schooling, mean	13.298	13.328	13.489	13.273
Unemployed, %	4.974	4.530	5.018	5.018
Disabled, %	11.491	13.854	11.553	11.223
Panel D. Demographic characteristics				
Female, %	50.351	50.104	50.875	50.317
Black, %	18.762	11.258	23.046	19.090
Hispanic/Latino, %	19.975	6.985	9.718	22.606
Native American, %	2.233	6.409	2.452	1.746
Asian, %	4.357	3.104	4.048	4.532
Immigrant, %	15.567	6.649	11.151	17.068
Married, %	49.108	50.266	49.397	48.947
Observations	6,190,223	5,84,389	6,02,275	5,003,559

The primary descriptive sample includes the experimental 2020 ACS with its supplied person's weight. Statistics are weighted using ACS person weights. Binary variables are percentages; observations are unweighted person-record counts. Other public coverage includes TRICARE/military or VA coverage. Coverage sources may overlap. FPL indicates federal poverty level.

Supplementary Table S1 presents a separate descriptive analysis of annual differences in any health insurance coverage across political state groups. In the political group analysis, the 2023 any-insurance estimates ranged from +1.187 points in red states to +2.178 points in swing states, with an estimate of +1.346 points in blue states (Supplementary Table S1). These estimates are descriptive calendar-year comparisons rather than DID estimates of Medicaid expansion.

### 3.2 Medicaid coverage in the full sample and policy-relevant subgroups

Table 2 and Figure 1 present the Medicaid DID estimates. In the full sample, the Oklahoma–Missouri estimate was +3.389 percentage points relative to the comparison states (95% CI, 1.024 to 5.754; exact randomization-inference *P* = 0.030). The corresponding North Carolina estimate was +3.332 percentage points (95% CI, 2.823 to 3.840; exact randomization-inference *P* = 0.091). The North Carolina *P*-value equaled the minimum attainable value among the 11 possible single-state treatment assignments.

**Table 2:** Weighted difference-in-differences estimates for Medicaid coverage in the full sample and subgroups.

Analytic sample	Oklahoma–Missouri (95% CI)	RI *P*	*N*	North Carolina (95% CI)	RI *P*	*N*
Full sample	+3.389^**^ (1.024, 5.754)	0.030	5,587,948	+3.332^*^ (2.823, 3.840)	0.091	5,605,834
Business owners	+4.322^**^ (2.779, 5.865)	0.015	474,897	+3.620^*^ (2.587, 4.652)	0.091	477,264
Below 138% FPL	+9.458^**^ (4.483, 14.432)	0.030	931,924	+8.007^*^ (6.991, 9.023)	0.091	926,885
Adults ages 18–35	+3.577^**^ (0.978, 6.176)	0.030	2,015,401	+3.763^*^ (3.083, 4.444)	0.091	2,018,501

Estimates and confidence intervals are percentage-point changes. Primary models include 2020 and state and year fixed effects. Models use PWGTP-weighted state-year rates and state-year population weights. CIs use state-clustered t-critical values. Significance symbols use exact randomization inference (RI): ^*^P < 0.10; ^**^P < 0.05; ^***^P < 0.01. N is the unweighted person-record count.

**Figure 1:**
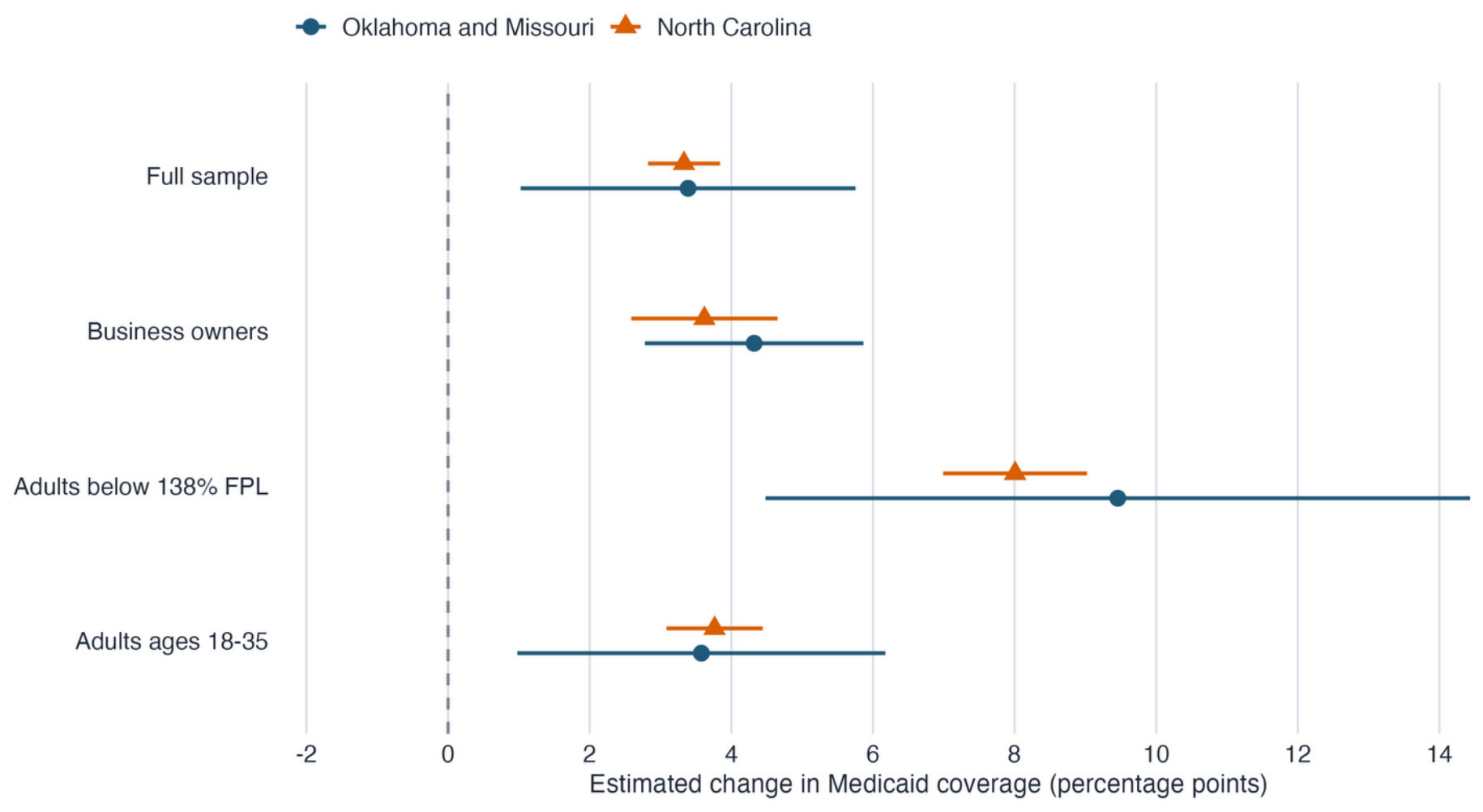
Difference-in-differences estimates of Medicaid coverage in the full sample and selected subgroups. Estimates are percentage-point changes among adults ages 18–64 using 2015–2024 ACS 1-year PUMS data, including the experimental 2020 ACS. Models use PWGTP-weighted state-year coverage rates, state-year population weights, and state and year fixed effects. Circles represent Oklahoma–Missouri, and triangles represent North Carolina. Horizontal lines indicate conventional state-clustered 95% confidence intervals. Exact randomization-inference P values are reported in Table 2. FPL, federal poverty level.

All subgroup estimates were positive. The largest estimates occurred among adults below 138% of the federal poverty level: +9.458 percentage points in Oklahoma–Missouri (95% CI, 4.483 to 14.432; exact randomization-inference *P* = 0.030) and +8.007 points in North Carolina (95% CI, 6.991 to 9.023; exact randomization-inference *P* = 0.091). Estimates for business owners and adults ages 18 to 35 ranged from +3.577 to +4.322 percentage points across the two treatment designs (Table 2 and Figure 1). Supplementary Table S5 reports the corresponding subgroup estimates after excluding 2020.

### 3.3 Changes in uninsurance and specific insurance sources

Table 3 and Figure 2 present the separate insurance-source estimates. In Oklahoma–Missouri, direct-purchase coverage declined by 1.968 percentage points relative to the comparison states (95% CI, −2.693 to −1.244; exact randomization-inference *P* = 0.015). The corresponding North Carolina estimate was −2.077 percentage points (95% CI, −3.008 to −1.146), but it was not supported by exact randomization inference (*P* = 0.182).

**Table 3:** Separate binary difference-in-differences estimates by insurance source.

Outcome	Oklahoma–Missouri (95% CI)	RI *P*	North Carolina (95% CI)	RI *P*
Medicaid	+3.389^**^ (1.024, 5.754)	0.030	+3.332^*^ (2.823, 3.840)	0.091
Uninsured	−0.863 (−2.115, 0.389)	0.348	−1.125 (−1.792, −0.459)	0.364
Employer-sponsored insurance	−0.264 (−1.952, 1.423)	0.788	+0.478 (0.029, 0.927)	0.636
Direct-purchase coverage	−1.968^**^ (−2.693, −1.244)	0.015	−2.077 (−3.008, −1.146)	0.182
Medicare	−0.084 (−0.403, 0.234)	0.788	−0.192 (−0.431, 0.047)	0.636
Other public coverage	0.000 (−0.211, 0.211)	1.000	+0.026 (−0.109, 0.161)	0.909
Multiple coverage	+0.102 (−0.241, 0.444)	0.758	+0.329 (−0.047, 0.705)	0.364

Estimates and confidence intervals are percentage-point changes. Each outcome is estimated separately, so coverage sources may overlap. Other public coverage includes TRICARE/military or VA coverage; Indian Health Service coverage is excluded. Primary models include 2020 and state and year fixed effects. CIs use state-clustered t critical values. Significance symbols use exact RI: ^*^P < 0.10; ^**^P < 0.05; ^***^P < 0.01.

**Figure 2:**
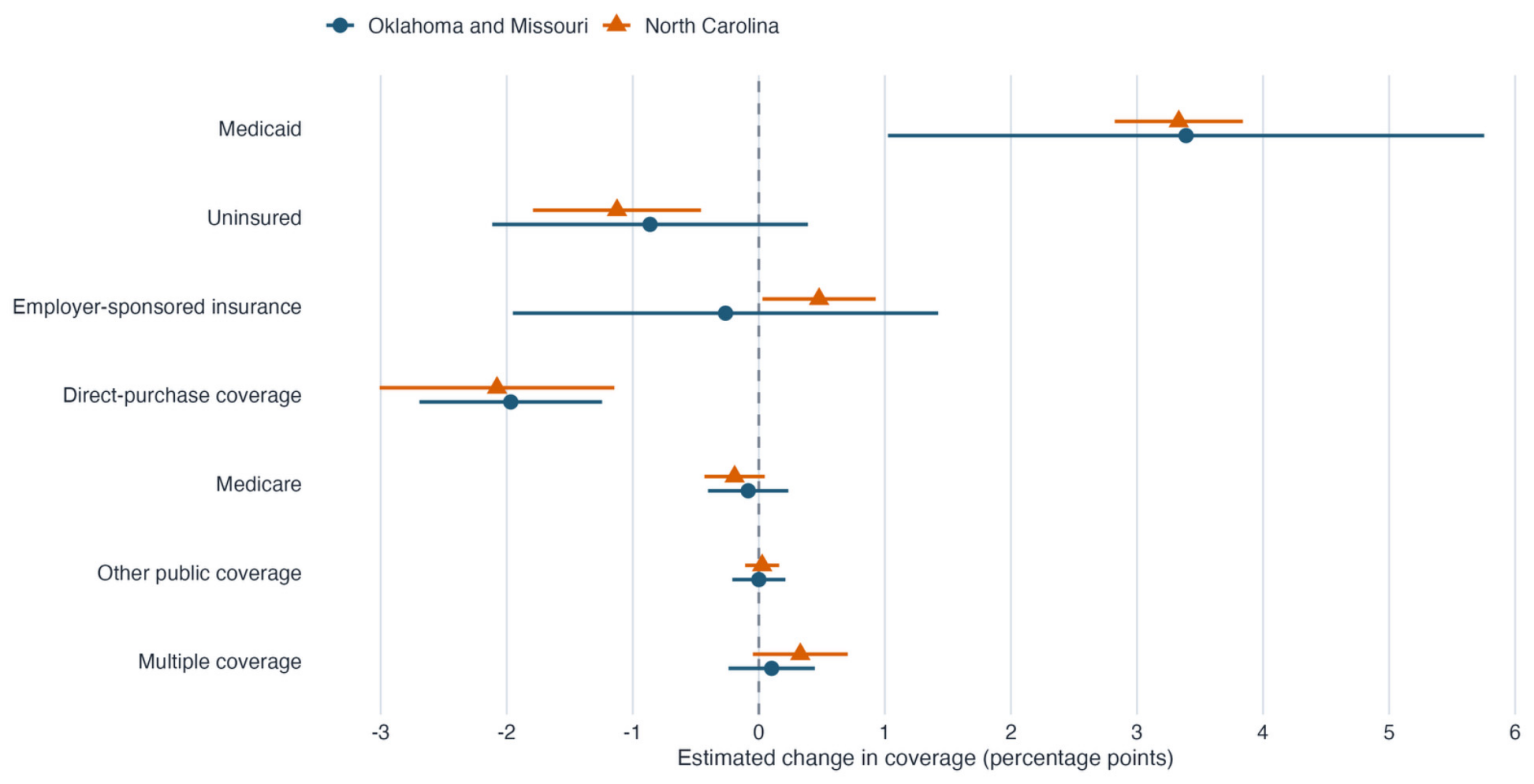
Difference-in-differences estimates by insurance source. Each outcome was estimated separately using 2015–2024 ACS 1-year PUMS data, including the experimental 2020 ACS; reported insurance sources may overlap. Models use PWGTP-weighted state-year coverage rates, state-year population weights, and state and year fixed effects. Horizontal lines indicate conventional state-clustered 95% confidence intervals. Other public coverage includes TRICARE/military or Department of Veterans Affairs coverage; Indian Health Service coverage is excluded. Exact randomization-inference P values are reported in Table 3.

The uninsurance estimates were −0.863 percentage points in Oklahoma–Missouri and −1.125 percentage points in North Carolina, with exact randomization–inference *P* values of 0.348 and 0.364, respectively. Changes in employer-sponsored insurance, Medicare, and other public coverage were also not supported by exact randomization inference. The adjusted multiple-coverage estimates were small: +0.102 percentage points in Oklahoma–Missouri (*P* = 0.758) and +0.329 points in North Carolina (*P* = 0.364). Descriptive multiple-coverage prevalence before and after expansion is reported in Supplementary Table S2, and the insurance-source estimates excluding 2020 are reported in Supplementary Table S4.

The supplementary HIMRKS decomposition showed different component patterns across the two treatment designs (Supplementary Table S3). In Oklahoma–Missouri, direct-purchase coverage without a premium subsidy declined by 0.883 percentage points (95% CI, −1.454 to −0.313; exact randomization-inference *P* = 0.045). In North Carolina, the larger component estimate was observed for subsidized Marketplace coverage, which declined by 1.031 percentage points (95% CI, −1.333 to −0.730; exact randomization-inference *P* = 0.091). The residual direct-purchase component was small in both designs, and estimates excluding 2020 had the same directions.

### 3.4 Timing, pre-expansion diagnostics, and medicaid unwinding

The conventional state-clustered joint tests of the pre-expansion event-study coefficients produced *P* = 0.061 with 2020 included and *P* = 0.172 with 2020 excluded for Oklahoma–Missouri. The corresponding North Carolina *P* values were 0.021 and 0.015, respectively (Table 4).

**Table 4:** Pre-expansion diagnostics and sensitivity analyses for Medicaid coverage.

Diagnostic or specification	Oklahoma–Missouri	Inference	North Carolina	Inference
Panel A. Primary and sensitivity estimates (95% CI)				
Primary; 2020 included	+3.389^**^ (1.024, 5.754)	0.030	+3.332^*^ (2.823, 3.840)	0.091
2020 excluded	+3.384^**^ (0.925, 5.843)	0.030	+3.296^*^ (2.760, 3.832)	0.091
Wisconsin excluded	+3.482^**^ (1.074, 5.890)	0.036	+3.377 (2.838, 3.916)	0.100
Restricted demographic covariates	+3.394^**^ (1.202, 5.587)	0.030	+3.374^*^ (2.904, 3.844)	0.091
Treatment-specific trend; 2020 included	+1.500 (−0.934, 3.935)	*P* = 0.202	+2.742 (2.489, 2.995)	*P* < 0.001
Treatment-specific trend; 2020 excluded	+0.241 (−2.777, 3.259)	*P* = 0.864	+2.656 (2.375, 2.937)	*P* < 0.001
Panel B. Joint test of pre-expansion event-study coefficients				
2020 included	F(5,11) = 2.977	*P* = 0.061	F(7,10) = 4.179	*P* = 0.021
2020 excluded	F(4,11) = 1.950	*P* = 0.172	F(6,10) = 4.760	*P* = 0.015
Panel C. Post-expansion timing (95% CI)				
2021–2022	+1.880^*^ (−0.648, 4.408)	0.076	–	–
2023–2024	+4.887^**^ (2.673, 7.102)	0.030	–	–
Late minus early	+3.007 (2.620, 3.395)	*P* < 0.001	–	–
2023 retained as transition	–	–	+3.332^*^ (2.823, 3.840)	0.091
2023 excluded	–	–	+3.428^*^ (2.895, 3.960)	0.091

Estimates and CIs are percentage-point changes. Exact RI *P* values appear without a *P* prefix. Values labeled P use conventional state-clustered inference, including the joint F tests, treatment-specific trend models, and late-minus-early contrast. Significance symbols use exact RI where available: ^*^P < 0.10; ^**^P < 0.05; ^***^P < 0.01. No symbol is shown for North Carolina after Wisconsin exclusion because its exact *P* value equals 0.100.

Supplementary Figure S1 presents the year-specific event-study coefficients and state-clustered 95% confidence intervals relative to 2019 for Oklahoma–Missouri and 2022 for North Carolina.

For Oklahoma–Missouri, the estimated change was +1.880 percentage points during the period 2021–2022 (exact randomization-inference *P* = 0.076) and +4.887 percentage points from 2023 to 2024 (*P* = 0.030). The late-minus-early contrast was +3.007 percentage points (95% CI, 2.620 to 3.395; conventional state-clustered *P* < 0.001). Exact randomization inference was not calculated for this contrast. For North Carolina, the estimate was +3.332 points when 2023 was retained as a transition year and +3.428 points when 2023 was excluded; both exact randomization-inference *P* values were 0.091 (Table 4 and Figure 3).

**Figure 3:**
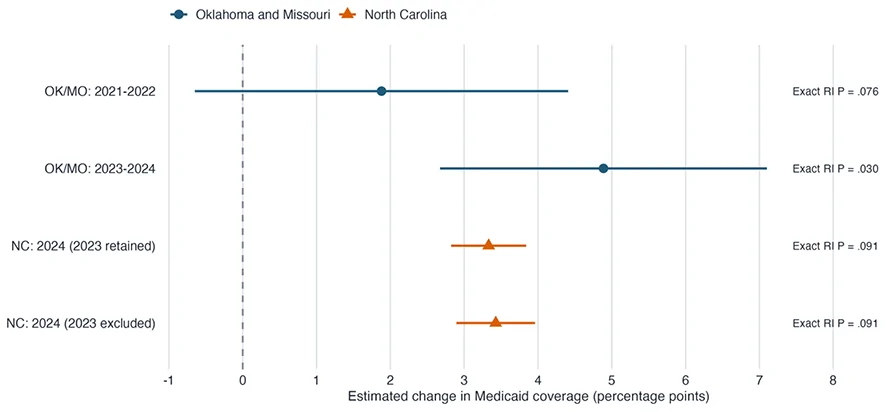
Timing of Medicaid coverage changes and North Carolina transition-year sensitivity. Estimates are percentage-point changes from population-weighted state-year models using 2015–2024 ACS data, including the experimental 2020 ACS, with state and year fixed effects. Oklahoma–Missouri estimates distinguish 2021–2022 from 2023–2024. North Carolina estimates compare specifications retaining or excluding the 2023 transition year; 2024 is its first full post-expansion year. Horizontal lines indicate conventional state-clustered 95% confidence intervals. Displayed P values use exact state-level randomization inference. Corresponding estimates are reported in Table 4.

The state-level unwinding measures differed across the treatment and comparison states. In 2023, the unweighted mean total-dis-enrollment measure was 42.668% in Oklahoma–Missouri and 39.181% across the comparison states, compared with 16.980% in North Carolina. The corresponding ex parte renewal measures were 54.178%, 42.703%, and 99.320%, respectively (Supplementary Table S6).

Adding each unwinding measure separately produced Oklahoma–Missouri estimates ranging from +3.322 to +3.385 percentage points, compared with +3.389 points in the baseline model; each adjusted estimate had an exact randomization-inference *P*-value of 0.030. With 2023 retained, the adjusted North Carolina estimates ranged from +2.906 to +3.098 points (*P* = 0.091 for each). When 2023 was excluded, the North Carolina estimates ranged from +0.643 to +3.550 points across the three unwinding specifications. In the total-disenrollment-adjusted model, the estimate was +0.643 points (95% CI, −0.293 to 1.580; exact randomization-inference *P* = 0.182; Table 5).

**Table 5:** Medicaid estimates adjusted for state-level unwinding measures.

Design and specification	Estimate (95% CI)	RI *P*
Panel A. Oklahoma–Missouri average, 2021–2024		
Baseline	+3.389^**^ (1.024, 5.754)	0.030
Adjusted for total disenrollment	+3.385^**^ (0.949, 5.821)	0.030
Adjusted for procedural disenrollment	+3.359^**^ (1.119, 5.600)	0.030
Adjusted for ex parte renewal	+3.322^**^ (0.644, 6.000)	0.030
Panel B. Oklahoma–Missouri, 2023–2024		
Baseline	+4.887^**^ (2.673, 7.102)	0.030
Adjusted for total disenrollment	+4.888^**^ (2.675, 7.101)	0.030
Adjusted for procedural disenrollment	+4.883^**^ (2.627, 7.138)	0.030
Adjusted for ex parte renewal	+4.985^**^ (2.779, 7.191)	0.030
Panel C. North Carolina, 2023 retained		
Baseline	+3.332^*^ (2.823, 3.840)	0.091
Adjusted for total disenrollment	+2.906^*^ (2.486, 3.327)	0.091
Adjusted for procedural disenrollment	+3.008^*^ (2.512, 3.504)	0.091
Adjusted for ex parte renewal	+3.098^*^ (2.275, 3.921)	0.091
Panel D. North Carolina, 2023 excluded		
Baseline	+3.428^*^ (2.895, 3.960)	0.091
Adjusted for total disenrollment	+0.643 (−0.293, 1.580)	0.182
Adjusted for procedural disenrollment	+2.001^*^ (1.298, 2.704)	0.091
Adjusted for ex parte renewal	+3.550^*^ (2.497, 4.603)	0.091

Primary models include 2020. Each unwinding measure enters a separate model. Total and procedural disenrollment are percentages of completed renewal outcomes; ex parte renewal is a percentage of renewed beneficiaries. Estimates and CIs are percentage-point changes. Significance symbols use exact RI: ^*^P < 0.10; ^**^P < 0.05; ^***^P < 0.01.

### 3.5 Sensitivity analyses and alternative counterfactuals

Excluding the experimental 2020 ACS produced estimates of +3.384 percentage points for Oklahoma–Missouri (95% CI, 0.925 to 5.843; exact randomization-inference *P* = 0.030) and +3.296 points for North Carolina (95% CI, 2.760 to 3.832; exact randomization-inference *P* = 0.091). Estimates obtained after removing Wisconsin or adding the restricted demographic covariates differed from the corresponding primary estimates by no more than 0.093 percentage points (Table 4).

Adding a separate linear trend for the treatment group reduced the Oklahoma and Missouri estimate to +1.500 percentage points with 2020 included (95% CI, −0.934 to 3.935; *P* = 0.202). Excluding 2020 reduced the estimate further to +0.241 percentage points (95% CI, −2.777 to 3.259; *P* = 0.864). The North Carolina estimates were +2.742 percentage points with 2020 included (95% CI, 2.489 to 2.995; *P* < 0.001) and +2.656 percentage points with 2020 excluded (95% CI, 2.375 to 2.937; *P* < 0.001). These confidence intervals and *P* values use conventional inference with standard errors clustered by state (Table 4).

Across conventional DID, traditional synthetic control, and synthetic DID specifications, the Oklahoma–Missouri estimates ranged from +3.328 to +3.479 percentage points, with assignment-based *P* values ranging from 0.015 to 0.045. The corresponding North Carolina estimates ranged from +2.953 to +3.332 percentage points, and all assignment-based *P* values equaled 0.091 (Table 6).

**Table 6:** Alternative counterfactual estimates for Medicaid coverage.

Method	2020 retained	RI *P*	2020 excluded	RI *P*
Panel A. Oklahoma–Missouri				
Conventional DID	+3.389^**^	0.030	+3.384^**^	0.030
Traditional synthetic control	+3.479^**^	0.030	+3.445^**^	0.045
Synthetic DID	+3.438^**^	0.015	+3.328^**^	0.015
Panel B. North Carolina				
Conventional DID	+3.332^*^	0.091	+3.296^*^	0.091
Traditional synthetic control	+2.994^*^	0.091	+2.998^*^	0.091
Synthetic DID	+3.011^*^	0.091	+2.953^*^	0.091

Entries are percentage-point changes. North Carolina retains 2023 as a transition year and estimates the 2024 gap. RI P values enumerate all 66 possible two-state assignments for Oklahoma–Missouri and all 11 possible single-state assignments for North Carolina. Synthetic methods reweight donor states and, for synthetic DID, pre-expansion periods. Significance symbols use RI P values: ^*^P < 0.10; ^**^P < 0.05; ^***^P < 0.01. Conventional state-clustered confidence intervals comparable with those for the DID models were not calculated for the synthetic estimators.

## 4 Discussion

### 4.1 Principal findings

This study examined changes in health insurance coverage following Medicaid expansions implemented well after the initial Affordable Care Act expansion period. Late Medicaid expansion was associated with higher Medicaid coverage in both treatment designs. Relative to contemporaneous changes in the comparison states, Medicaid coverage increased by 3.389 percentage points in Oklahoma–Missouri and by 3.332 percentage points in North Carolina. The Oklahoma–Missouri estimate was supported by exact randomization inference (*P* = 0.030). For North Carolina, the observed estimate was the most extreme among the 11 possible single-state treatment assignments. However, the minimum attainable exact *P* value was 0.091, so this procedure could not reject the null hypothesis at the 5% significance level. This limitation reflects the small number of possible treatment assignments.

The largest point estimates occurred among adults with family income below 138% of the federal poverty level, with increases of 9.458 percentage points in Oklahoma–Missouri and 8.007 percentage points in North Carolina. This concentration is consistent with the eligibility structure of ACA Medicaid expansion because this income group most closely corresponds to the population newly eligible under the policy. The magnitude of these estimates is also broadly consistent with the evaluation of Virginia's 2019 expansion by Lyu and Wehby, who reported Medicaid gains of approximately 9–11 percentage points among adults below 100% of the federal poverty level and still larger gains among adults between 100% and 138% of the federal poverty level (10). Differences in the study period, states, samples, and methods prevent direct numerical equivalence, but both studies indicate that the largest coverage response occurred among adults closest to the expansion eligibility threshold.

Positive Medicaid estimates were also observed among business owners and adults ages 18–35. The analysis of adults ages 18 to 35 used an age restriction without conditioning inclusion on observed labor supply. These estimates indicate that the positive coverage association was not confined to the full working-age sample. Nevertheless, the subgroup coefficients were estimated separately, and formal differences between subgroup estimates were not tested. The results therefore should not be interpreted as demonstrating that one subgroup experienced a statistically larger response than another.

The coverage-source results show that growth in Medicaid coverage should not be interpreted as equivalent to a one-for-one reduction in uninsurance. In both treatment designs, the Medicaid increase was larger than the estimated decline in uninsurance, and the uninsurance estimates were not supported by exact randomization inference. The observed Medicaid growth may therefore reflect a combination of reductions in uninsurance, changes in other insurance sources, and changes in overlapping coverage rather than only previously uninsured adults obtaining Medicaid.

### 4.2 Insurance-source changes and crowd-out

Separating insurance sources substantially narrows the interpretation of crowd-out. In Oklahoma–Missouri, direct-purchase coverage declined by 1.968 percentage points, and this decline was supported by exact randomization inference (*P* = 0.015). In contrast, there was no assignment-based evidence of a corresponding decline in employer-sponsored insurance. The Oklahoma–Missouri findings are therefore consistent with partial substitution away from direct-purchase coverage, rather than broad displacement of private insurance or employer-sponsored coverage.

In Oklahoma and Missouri, the absolute value of the direct purchase coefficient was 58.1% of the Medicaid coefficient. Lennon reported crowding out estimates of 43% among adults with low incomes and 56% among working adults within that population (16). The present ratio is numerically higher, but the figures describe different populations, insurance sources, and expansion periods. Lennon examined broader private insurance during earlier expansions. The present analysis examines direct purchase coverage among adults ages 18 to 64 during later expansions. The 58.1% ratio compares two separately estimated coefficients and has no separate confidence interval. It does not identify an individual transition rate or an independently estimated crowding out parameter.

More broadly, previous ACS studies have produced mixed evidence concerning private-coverage substitution. Lyu and Wehby found substantial Medicaid gains following Virginia's 2019 expansion but no discernible change in private coverage (10). Other studies of earlier expansion cohorts reported relative reductions in employer-sponsored and directly purchased insurance among particular low-income populations (29, 30). This study falls between these findings: it identifies a decline in direct-purchase coverage in Oklahoma–Missouri but does not find evidence of employer-sponsored insurance displacement. This distinction demonstrates why Medicaid, employer-sponsored insurance, and direct-purchase coverage should not be combined into a single residual insurance category.

The supplementary HIMRKS decomposition provides additional context. In Oklahoma–Missouri, the strongest assignment-based evidence was for a decline in direct-purchase coverage without a Marketplace premium subsidy. The decline in subsidized Marketplace coverage was smaller and received weaker assignment-based support. The no-subsidy category may include both unsubsidized Marketplace plans and coverage purchased outside the Marketplace, so it cannot be interpreted as a pure off-Marketplace category. Nevertheless, its decline is more consistent with conventional private-coverage substitution than a decline confined to subsidized Marketplace coverage would be.

North Carolina also had a negative direct-purchase estimate, and its conventional confidence interval excluded zero. However, the full-period exact randomization-inference *p*-value was 0.182. The North Carolina result therefore does not provide conclusive assignment-based evidence of private-insurance crowd-out. Moreover, in the shorter HIMRKS analysis, the larger component decline occurred in subsidized Marketplace coverage. A simultaneous Medicaid increase and subsidized Marketplace decline may be consistent with changes at the Marketplace–Medicaid eligibility boundary rather than economically equivalent displacement of employer-sponsored or unsubsidized private insurance. As HIMRKS is available only from 2019 onward and the ACS does not follow the same individuals over time, this component analysis should be treated as supplementary evidence rather than proof that individuals moved from one insurance source to another.

Multiple coverage was present before and after expansion (Supplementary Table S2). The adjusted estimates were small and were not supported by exact randomization inference in either treatment design. This outcome measures the prevalence of any combination of two or more insurance sources. It does not isolate Medicaid combined with another source or track individual changes in coverage. The results therefore provide no clear evidence of a change in overall multiple coverage. They do not rule out changes in particular insurance combinations.

### 4.3 Policy lag, Medicaid unwinding, and alternative counterfactuals

In Oklahoma and Missouri, the estimated Medicaid increase was 1.880 percentage points during 2021 to 2022 and 4.887 percentage points during 2023 to 2024. The corresponding exact randomization inference *P*-values were 0.076 and 0.030. The latter estimate exceeded the earlier estimate by 3.007 percentage points. The test of this contrast used conventional inference with standard errors clustered by state. Exact randomization inference was not calculated for the contrast, and its precision requires caution with only two treated states. Both expansions began during 2021, so the early period included partial exposure. Gradual enrollment and implementation may also contribute to larger estimates in later years. The timing pattern alone cannot distinguish these explanations.

Medicaid unwinding coincided with the later period. Differences in disenrollment between treatment and comparison states could contribute to the estimated coverage differences. The descriptive indicators did not show consistently lower disenrollment in Oklahoma and Missouri than in the comparison states. The two treatment states also differed from each other. Separate adjustments for total disenrollment, procedural disenrollment, and ex parte renewal produced estimates of 4.883 to 4.985 percentage points for 2023 to 2024, close to the baseline estimate of 4.887 percentage points. These adjustments capture selected aspects of renewal activity. They do not fully account for differences in outreach, reporting, administration, economic conditions, or other insurance market changes. The results remain consistent with a gradually increasing coverage association after expansion, but they do not establish delayed enrollment as the mechanism.

North Carolina requires greater caution. Its first complete year after expansion coincided with Medicaid unwinding. Its reported disenrollment and ex parte renewal measures also differed substantially from those of the comparison states. With 2023 retained as a transition year, the estimate remained positive after each unwinding adjustment. Excluding 2023 increased sensitivity to the choice of unwinding measure. Adjustment for total disenrollment reduced the estimate to 0.643 percentage points, with a confidence interval containing zero and an exact randomization inference *P*-value of 0.182. The available data cannot clearly separate the expansion association from differences in enrollment administration.

The diagnostics also raised concerns about trends before expansion. For Oklahoma and Missouri, the joint tests produced *P* = 0.061 with 2020 included and *P* = 0.172 with 2020 excluded. The corresponding North Carolina *P* values were 0.021 and 0.015. These conventional tests indicate a clearer concern about parallel trends in North Carolina.

Alternative counterfactual models produced different results. Adding a separate linear trend for the treatment group reduced the Oklahoma and Missouri estimate to 1.500 percentage points with 2020 included and 0.241 percentage points with 2020 excluded. Both confidence intervals included zero. Traditional synthetic control and synthetic DID produced positive estimates close to the conventional DID estimates in both treatment designs. These approaches use different assumptions to construct coverage paths in the absence of expansion. Agreement between the conventional and synthetic estimates provides supporting evidence, but it does not resolve the concerns about trends before expansion.

The primary estimates changed little after excluding the experimental 2020 ACS, removing Wisconsin, or adjusting for the restricted demographic covariates. However, the Oklahoma and Missouri estimates remained sensitive to the assumed linear trend. North Carolina retained concerns about trends before expansion and the overlap between expansion and unwinding. These findings support cautious interpretation of the estimated coverage associations.

### 4.4 Policy implications

Late Medicaid expansion was associated with increased Medicaid coverage in the states examined. The largest point estimates occurred among adults below 138% of the federal poverty level. This pattern is consistent with the policy's focus on adults with low incomes. The findings extend evidence on Medicaid coverage beyond the initial ACA expansion period and into a different insurance and administrative environment.

The larger estimates in later years support continued evaluation beyond the initial implementation period. Evaluations should distinguish partial years of exposure from complete years after expansion and report changes across successive periods. Additional observations after 2024 are needed to assess the persistence of the Oklahoma and Missouri association and coverage patterns beyond North Carolina's first complete year.

Coverage monitoring should report Medicaid, uninsurance, employer-sponsored insurance, direct purchase coverage, and multiple coverage separately. An increase in Medicaid coverage does not necessarily represent an equivalent reduction in uninsurance. The clearest offsetting change occurred in direct purchase coverage in Oklahoma and Missouri. The analysis did not find corresponding evidence of a decline in employer-sponsored insurance under exact randomization inference. Changes between Medicaid and subsidized Marketplace coverage may reflect coordination between eligibility rules and premium subsidies. Such changes have different welfare and fiscal implications from the displacement of employer-sponsored or unsubsidized private insurance.

### 4.5 Strengths and limitations

This study has several strengths. (1) It uses 2015–2024 ACS 1-year PUMS data, providing multiple pre-expansion observations and extending through North Carolina's first complete post-expansion year. (2) It examines specific, potentially overlapping insurance sources and policy-relevant subgroups while avoiding potentially post-treatment individual covariates in the principal models. (3) It supplements conventional state-clustered confidence intervals with exact state-level randomization inference and evaluates the findings using pretrend diagnostics, alternative time and comparison-group specifications, Medicaid unwinding measures, and synthetic counterfactual methods.

This study also has several limitations. (1) Medicaid expansion was not randomly assigned, and state adoption was shaped by political and institutional factors (31, 32). The pre-expansion diagnostics raised concerns, particularly for North Carolina, and the Oklahoma–Missouri estimate was sensitive to the inclusion of a treatment-specific trend. The findings should therefore be interpreted as associations rather than definitive causal effects, and the additional analyses cannot eliminate all possible time-varying confounding, such as differences in Medicaid unwinding. (2) Each design contains only one or two treated states. Consequently, the exact randomization–inference distributions are discrete, and North Carolina's smallest attainable exact *P*-value is 0.091. North Carolina also contributes only one complete post-expansion year. (3) The ACS is a repeated cross-sectional survey and does not follow individuals over time. It therefore cannot establish that particular respondents moved from direct-purchase coverage to Medicaid; the Oklahoma–Missouri results indicate a population-level pattern consistent with partial crowd-out rather than directly observed insurance transitions. (4) The analysis covers only three late-expansion states and relies on annual, self-reported insurance measures. Although the findings were similar after excluding the experimental 2020 ACS, pandemic-era disruptions, variation in state-reported unwinding data, and the limited follow-up period may affect measurement and generalizability.

Medicaid expansions implemented after 2020 were associated with increased Medicaid coverage, particularly among adults below 138% of the federal poverty level. The Oklahoma and Missouri findings were consistent with partial substitution away from direct purchase coverage. The estimated Medicaid increase was also larger in later years. Concerns about trends before expansion, the small number of treated states, and North Carolina's distinctive unwinding experience limit causal interpretation. Additional years of observation can strengthen the assessment of the persistence and timing of these coverage changes.

## Data Availability

The data analyzed in this study are publicly available from the U.S. Census Bureau American Community Survey Public Use Microdata Sample: https://www.census.gov/programs-surveys/acs/microdata.html, and the Centers for Medicare & Medicaid Services (CMS) State Medicaid and CHIP Eligibility Processing Data, available through Data.Medicaid.gov: https://data.medicaid.gov/datasets.
