# Supplementary material for: Late Medicaid expansion was associated with higher Medicaid coverage: source-specific evidence from Oklahoma, Missouri, and North Carolina
Source: Front Public Health. 2026 Oct 8;14:1942538. doi: 10.3389/fpubh.2026.1942538 (PMC13686982; doi:10.3389/fpubh.2026.1942538)
Supplement: Supplementary file 1 [file Table_1.pdf]

Supplementary Methods. Descriptive analysis of insurance coverage by political state group

A separate national extract from the 2020 to 2024 ACS 5-year PUMS was used for the descriptive analysis in Supplementary Table S1 (1). The extract included an approximately 10% household subsample and 711,135 person records with complete data. It differed from the annual ACS 1-year PUMS files used in the primary analyses and was not used to estimate changes associated with late Medicaid expansion.

States were classified using presidential voting patterns in 2020 and 2024. Arizona, Georgia, Michigan, Nevada, North Carolina, Pennsylvania, and Wisconsin were classified as swing states. Among the remaining states, blue states supported the Democratic presidential candidate in both elections, and red states supported the Republican presidential candidate in both elections (2, 3).

Separate linear probability models estimated annual differences in any health insurance coverage within each political group, with 2020 as the reference year. Models used the ACS person weight, PWGTP, and adjusted for years of schooling, personal income, business ownership, age, age squared, female sex, Black race, Hispanic or Latino ethnicity, Native American race, immigrant status, unemployment, disability, and marital status. Standard errors were clustered by state. These estimates describe annual coverage patterns within political groups. They do not identify effects of partisanship or Medicaid expansion.

Supplementary Table S1. Adjusted year differences in any health insurance coverage by political state group, 2020–2024

Year	Red states (95% CI)	Blue states (95% CI)	Swing states (95% CI)
2021	+0.362 (-0.394, 1.119)	+0.575*** (0.162, 0.987)	+0.619 (-0.702, 1.940)
2022	+0.955 (-0.261, 2.171)	+1.287*** (0.531, 2.042)	+1.287 (-0.280, 2.854)
2023	+1.187** (0.193, 2.181)	+1.346*** (0.836, 1.855)	+2.178*** (0.972, 3.384)
2024	+1.783*** (0.733, 2.833)	+1.154** (0.249, 2.060)	+1.090 (-0.896, 3.076)
Observations	269,139	313,329	128,667
R^2	0.126	0.088	0.103

Note: Estimates are percentage point differences relative to 2020. Models use ACS person weights and adjust for the covariates listed in Supplementary Methods. Confidence intervals and

significance symbols use conventional inference with standard errors clustered by state. One asterisk indicates P<.10, two indicate P<.05, and three indicate P<.01. Political groups are defined in Supplementary Methods. These estimates do not identify effects of partisanship or Medicaid expansion.

Supplementary Table S2. PWGTP-weighted prevalence of multiple insurance coverage before and after Medicaid expansion

Treatment design	Group	Pre/Transition, %	Post, %	Change
Oklahoma-Missouri	Treatment states	7.836 (2015-2020)	8.727 (2021-2024)	+0.891
Oklahoma-Missouri	Comparison states	7.640 (2015-2020)	8.408 (2021-2024)	+0.768
North Carolina	Treatment state	8.989 (2015-2023)	9.841 (2024)	+0.852
North Carolina	Comparison states	7.903 (2015-2023)	8.401 (2024)	+0.498

Note: Multiple coverage was defined as reporting at least two of the six comprehensive insurance sources represented by HINS1–HINS6. Percentages are person-level estimates weighted using the ACS person weight (PWGTP). Changes are percentage-point differences. The primary specification includes the 2020 ACS using its supplied experimental person weights. North Carolina implemented expansion on December 1, 2023; 2023 was therefore treated as a transition year, and 2024 was the first full post-expansion year. These descriptive changes are not adjusted DID estimates; adjusted estimates appear in Table 3

Supplementary Table S3. Direct-purchase coverage decomposition using the ACS Marketplace-subsidy indicator, 2019–2024

Outcome	Oklahoma–Missouri (95% CI)	RI P	North Carolina (95% CI)	RI P
**Panel A. 2019-2024, 2020 retained**				
Medicaid	+3.285** (0.895, 5.674)	.030	+3.109* (2.779, 3.438)	.091
All direct-purchase coverage	-1.589** (-2.169, -1.009)	.015	-1.858* (-2.595, -1.122)	.091

Subsidized Marketplace coverage	-0.570* (-0.833, -0.308)	.091	-1.031* (-1.333, -0.730)	.091
Direct purchase without a premium subsidy	-0.883** (-1.454, -0.313)	.045	-0.824 (-1.283, -0.365)	.364
Residual direct purchase coded HIMRKS=0	-0.135* (-0.301, 0.030)	.076	-0.003 (-0.022, 0.016)	1.000
**Panel B. 2019-2024, 2020 excluded**				
Medicaid	+3.155** (0.292, 6.018)	.030	+2.984* (2.639, 3.329)	.091
All direct-purchase coverage	-1.691** (-2.426, -0.957)	.030	-1.679 (-2.377, -0.981)	.182
Subsidized Marketplace coverage	-0.508 (-0.797, -0.219)	.227	-0.977* (-1.254, -0.701)	.091
Direct purchase without a premium subsidy	-1.059* (-1.685, -0.433)	.061	-0.694 (-1.149, -0.240)	.455
Residual direct purchase coded HIMRKS=0	-0.124* (-0.261, 0.013)	.076	-0.007 (-0.027, 0.012)	.909

*Note: Among respondents reporting direct-purchase coverage (HINS2=1), HIMRKS=1 identifies coverage with a premium subsidy, HIMRKS=2 identifies direct-purchase coverage without a premium subsidy, and the residual measure requires HINS2=1 and HIMRKS=0. Estimates and CIs are percentage-point changes. Significance symbols use exact RI: *P<.10; **P<.05; ***P<.01. The component estimates do not identify individual coverage transitions.*

Supplementary Table S4. Insurance-source robustness estimates excluding the experimental 2020 ACS

Outcome, 2015-2024 excluding 2020	Oklahoma–Missouri (95% CI)	RI P	North Carolina (95% CI)	RI P
Medicaid	+3.384** (0.925, 5.843)	.030	+3.296* (2.760, 3.832)	.091
Uninsured	-0.677 (-1.875, 0.521)	.455	-1.110 (-1.787, -0.432)	.455

Employer-sponsored insurance	-0.403 (-2.327, 1.520)	.652	+0.436 (-0.040, 0.912)	.636
Direct-purchase coverage	-2.064** (-2.856, -1.272)	.015	-2.014 (-2.952, -1.076)	.273
Medicare	-0.062 (-0.429, 0.305)	.833	-0.193 (-0.433, 0.047)	.636
Other public coverage	-0.047 (-0.260, 0.165)	.803	+0.027 (-0.108, 0.162)	.909
Multiple coverage	+0.026 (-0.376, 0.427)	.939	+0.347 (-0.023, 0.717)	.455

*Note: Each outcome is estimated separately, so coverage sources may overlap. Estimates and CIs are percentage-point changes. Models exclude 2020 and include state and year fixed effects. Significance symbols use exact RI: *P<.10; **P<.05; ***P<.01.*

Supplementary Table S5. Medicaid robustness estimates by analytic sample after excluding the experimental 2020 ACS

Analytic sample, 2020 excluded	Oklahoma–Missouri (95% CI)	RI P	N	North Carolina (95% CI)	RI P	N
Full sample	+3.384** (0.925, 5.843)	.030	5,168,721	+3.296* (2.760, 3.832)	.091	5,197,271
Business owners	+4.247** (2.768, 5.726)	.015	439,711	+3.740* (2.730, 4.750)	.091	442,975
Below 138% FPL	+9.396** (4.249, 14.544)	.030	866,460	+8.046* (6.989, 9.104)	.091	863,661
Adults ages 18-35	+3.627** (0.925, 6.329)	.030	1,861,158	+3.720* (3.002, 4.438)	.091	1,868,145

*Note: Estimates and CIs are percentage-point changes. Models exclude 2020 and include state and year fixed effects. Significance symbols use exact RI: *P<.10; **P<.05; ***P<.01. N is the unweighted person-record count.*

Supplementary Table S6. State-level Medicaid unwinding measures, 2023–2024

State	Months reported/possible	Total disenrollment (%)	Procedural disenrollment (%)	Ex parte renewal (%)
**Panel A. 2023 (April–December)**				
**Treatment states**				
Oklahoma	9/9	55.508	45.814	30.712
Missouri	7/9	29.828	23.350	77.644
North Carolina	7/9	16.980	14.599	99.320
**Comparison states**				
Alabama	7/9	32.806	28.364	52.975
Florida	9/9	37.985	24.700	34.506
Georgia	8/9	49.347	37.679	60.231
Kansas	9/9	41.596	27.421	46.110
Mississippi	7/9	26.883	20.163	29.976
South Carolina	8/9	40.279	27.225	49.936
Tennessee	7/9	38.345	29.565	71.421
Texas	8/9	59.600	41.382	10.177
Wisconsin	7/9	45.458	27.709	20.605
Wyoming	9/9	19.510	14.368	51.091

Supplementary Table S6 (continued)

State	Months reported/possible	Total disenrollment (%)	Procedural disenrollment (%)	Ex parte renewal (%)
**Panel B. 2024 (January–August)**				

**Treatment states**				
Oklahoma	8/8	40.511	31.637	46.273
Missouri	8/8	29.943	25.185	82.391
North Carolina	8/8	9.078	7.511	99.579
**Comparison states**				
Alabama	8/8	31.679	24.535	56.826
Florida	8/8	35.482	21.810	34.397
Georgia	8/8	33.813	19.210	73.376
Kansas	8/8	21.446	8.127	41.198
Mississippi	8/8	18.070	13.377	34.552
South Carolina	8/8	31.682	19.146	79.532
Tennessee	8/8	39.038	28.532	71.366
Texas	8/8	29.518	17.804	16.209
Wisconsin	8/8	29.345	14.870	49.590
Wyoming	8/8	29.914	21.824	60.181

Note: Source: Centers for Medicare & Medicaid Services State Medicaid and CHIP Eligibility Processing Data. Annual measures use sums of monthly counts over the specified reporting windows. Total and procedural disenrollment are percentages of completed renewal outcomes; ex parte renewal is a percentage of renewed beneficiaries. Months reported/possible gives months with nonmissing outcome data over expected months in each window. CMS notes that North Carolina reports outcomes only for initiated renewals scheduled for completion and does not include renewals that the state was unable to initiate by the end of the reporting month; its values should therefore be compared cautiously.

Supplementary Figure S1. Event-study estimates of Medicaid coverage in Oklahoma–Missouri and North Carolina, 2015–2024.

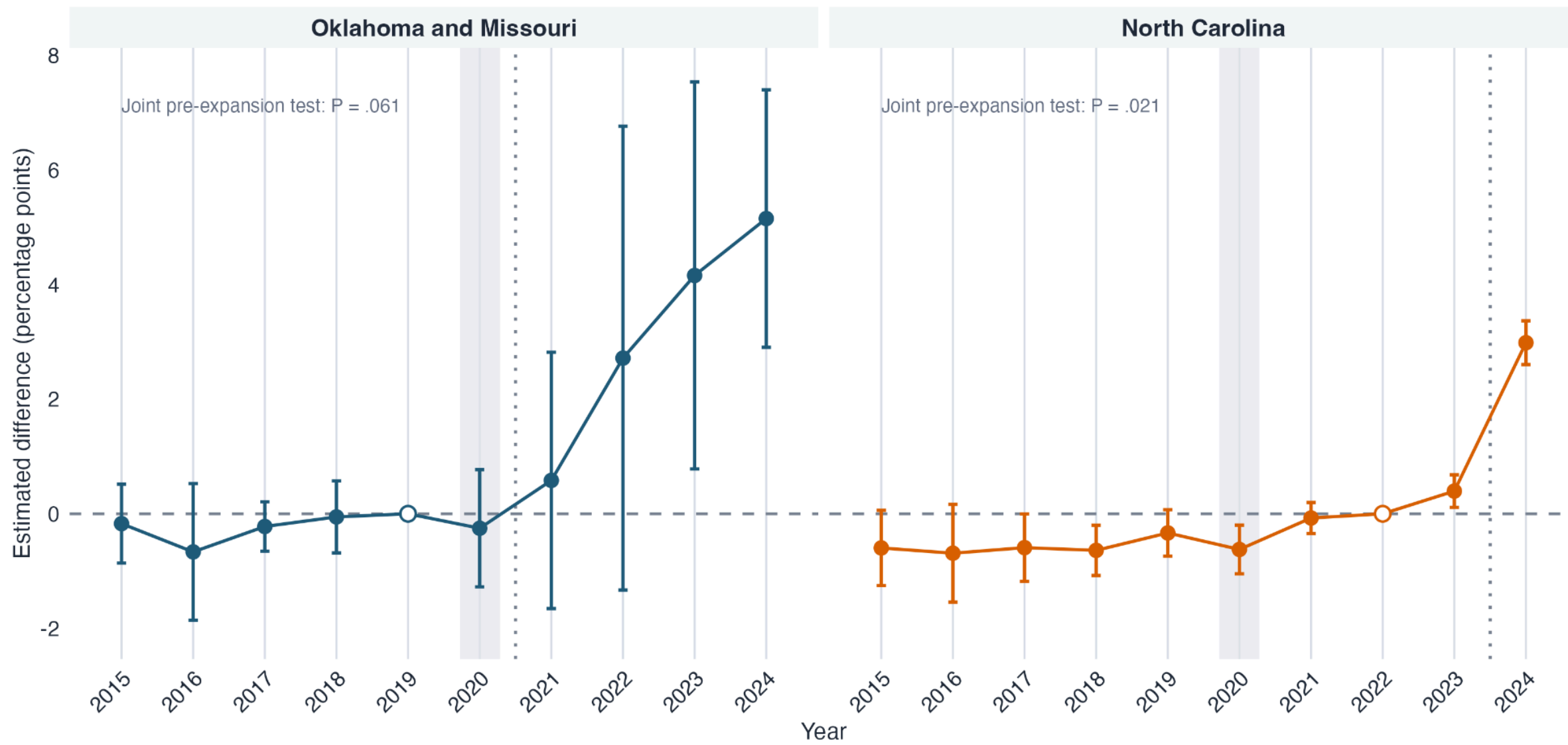

Points show treatment-by-year coefficients in percentage points from population-weighted state-year models with state and year fixed effects. Error bars indicate conventional state-clustered 95% confidence intervals. Open circles identify the omitted reference years: 2019 for Oklahoma–Missouri and 2022 for North Carolina. Gray shading identifies the experimental 2020 ACS. Dotted vertical lines mark the boundary before the first post-coded year: 2021 for Oklahoma–Missouri and 2024 for North Carolina. North Carolina's 2023 observation is retained as a transition year and excluded from its joint pre-expansion test. Conventional joint pre-expansion tests yielded P = .061 for Oklahoma–Missouri and P = .021 for North Carolina.

Supplementary References

1. U.S. Census Bureau. 2020–2024 American Community Survey 5-year Public Use Microdata Sample person file [dataset]. Washington, DC: U.S. Census Bureau (2026). Available at: https://www.census.gov/programs-surveys/acs/microdata/access.html (Accessed September 12, 2026).

2. Federal Election Commission. Federal elections 2020: election results for the U.S. President, the U.S. Senate and the U.S. House of Representatives (2021). Available at: https://www.fec.gov/resources/cms-content/documents/federalelections2020.pdf (Accessed September 12, 2026).

3. Federal Election Commission. Official 2024 presidential general election results (2025). Available at: https://www.fec.gov/documents/5644/2024presgeresults.pdf (Accessed September 12, 2026).
